# Deletion in 1p36.33-p36.32 is associated with pancytopenia: a case report

**DOI:** 10.1186/s12920-023-01723-4

**Published:** 2023-11-09

**Authors:** Huanhuan Yang, Jun Huang, Hao Zheng, Yunfan Zhang, Yuanzhen Zhang, Wei Liu, Jinrong Wu, Xiaobin Chen, Jinfeng Lin, Yanna Ni, Xiaojing Nie

**Affiliations:** 1 Fuzong Clinical Medical College of Fujian Medical University, Fuzhou, China; 2 Department of Pediatrics, The 900th Hospital of Joint Logistic Support Force, PLA, Fuzhou, China; 3 Department of Pediatrics, Fujian Union Hospital, Fuzhou, China; 4 Department of Pediatrics, Affiliated Dongfang Hospital, Xiamen University, Fuzhou, China

**Keywords:** 1p36 deletion, IRH, Haploinsufficiency, Genotype-phenotype correlation, Copy number variation, Gene sequencing

## Abstract

**Background:**

1P36 deletion syndrome is recognized as the most common terminal microdeletion syndrome in humans, characterized by early developmental delay and consequent intellectual disability, seizure disorder, and distinctive facial features. Variable deletion locations may attributed to phenotypic variability. However, the abnormal phenotypes of hematology are rarely reported in 1P36 deletion syndrome patients.

**Case presentation:**

We present a case of postnatal intellectual disability accompanied by pancytopenia. Copy number variation analysis revealed a pathogenic deletion in 1p36.331p36.32 with a deletion size of 2.21 Mb. Following successful treatment with glucocorticoids, the patient was diagnosed with immuno-related hemocytopenia (IRH).

**Discussion:**

The patient experienced IRH, an uncommon characteristic of 1p36 deletion syndrome. The deletion fragment of 1p36.33-p36.32, particularly the loss of GNB1 gene, has been associated with the development of pancytopenia. Genotype-phenotype correlations are valuable in identifying the genes responsible for various clinical characteristics of the syndrome by associating phenotypic variation with specific genes located within the chromosome deletion region. Genome sequencing is recommended in cases where clinical manifestations indicate the presence of a genetic disorder but pose diagnostic challenges.

## Introduction

The incidence of chromosome 1p36 deletion is estimated to be approximately 1 in every 5,000 live births. Therefore, 1p36 deletion syndrome is one of the most common terminal deletions in humans. The primary manifestations include developmental and intellectual disabilities, visual impairments, epileptic seizures, hypotonia, characteristic facial features, as well as cardiovascular abnormalities [1, 2]. The phenotypic diversity of Chromosome 1p36 deletion syndrome arises from variations in the size and location of the deleted fragments, resulting in distinct haploinsufficient effects. The hematologic disorders associated with 1p36 deletion syndrome have primarily been reported as hematologic neoplasms. In 2009, Katzenberger et al. reported a unified genetic alteration (deletion of 1p36) in 29 cases of Follicular lymphoma, suggesting that del(1p36) may constitute a primary aberration of this specific tumor [3]. Lahortiga et al. analyzed the location of the breakpoints (BPs) on 1p36 of 26 patients with hematological neoplasia, discovering clustered BPs in a 2.5 Mb region located between 1p36.32 and the telomere in 14 out of 26 cases, which was included in the 10.5 Mb region defined for 1p36 deletion syndrome [4]. The current knowledge does not include any reports on the association between 1P36 deletion syndrome and IRH.

## Case presentation

The patient was a 4-year and 5-month-old male. He was admitted to our hospital on October 19, 2021, presenting with a complaint of pancytopenia persisting for over one month. The patient was admitted to a local hospital on August 24, 2021, due to the presence of petechiae on both lower extremities for 14 days. The laboratory tests revealed pancytopenia, characterized by a leukocyte count of 0.67 × 10^9^/L, a hemoglobin level of 112.0 g/L, and a platelet count of 1.0 × 10^9^/L. The patient underwent platelet transfusion and received etamsylate to improve hemorrhage. There was no significant improvement observed in the blood cell count. Consequently, a bone marrow aspiration was performed, revealing notable proliferation of granular lineage and impaired maturation of megakaryocytes. The patient received treatment with gamma globulin and glucocorticoids from September 19, 2021, to September 27, 2021. The blood cell count revealed an increase in leukocyte count to 5.47 × 10^9/^L, a hemoglobin count of 121 g/L, and a platelet count of 42 × 10^9^/L. The blood cell count was retested on October 19, 2021, revealing a decrease in the leukocyte count to 0.9 × 10^9^/L, a hemoglobin level of 86.0 g/L and a platelet count of 39 × 10^9^/L. Subsequently, the patient was transferred to our hospital.

The child was delivered via cesarean section at full term and had a birth weight of 2600 g. He required ventilatory support due to the presence of meconium aspiration syndrome. He started to experience convulsion at the age of 5 months, characterized by unilateral limb twitching. The Electroencephalograph (EEG) and cranial magnetic resonance imaging (MRI) did not reveal any abnormalities. At the age of 2 years and one month, a cranial magnetic resonance examination was performed due to intellectual disability, revealing a small round abnormal signal in the right frontal lobe and possibly enlargement of the Vascular clearance. The correction of strabismus was performed when the patient reached the age of 3 years. His non-consanguineous-mating parents and two sisters were both healthy.

The patient’s growth was significantly delayed. He could not lift his head at the age of 3 months, could not turn over and sit alone until 10 months, or could not walk until the age of 2 years and four months. He could run unstably at admission and could not do single-leg jump. It wasn’t until he was two that he could consciously call out Mom and Dad. The patient presented with an inability to articulate coherent sentences or comply with basic instructions upon admission.

The height measured 105 cm, the weight recorded 23 kg, the body temperature registered 36.3℃, the pulse rate was 100/min, and the blood pressure was 91/52 mmHg. The physical examination showed no significant abnormalities.

The laboratory tests revealed liver dysfunction with aspartate aminotransferase (AST) levels of 55.1 U/L and alanine aminotransferase(ALT) level of 94.2 U/L. The results of multiple pathogen tests (including Cytomegalovirus, Epstein-Barr virus, TORCH, Enterovirus, etc.) and auto-immune diseases assays (such as anti-nuclear antibody, anticardiolipin antibody, and anti-dsDNA antibody) were all negative. Coomb’s test was negative. The abdominal computerized tomography revealed a mildly enlarged liver. The cranial MRI revealed areas of softening foci adjacent to the anterior horn of the right ventricle. The bone marrow examination revealed proliferative anemia, thrombocytopenia, and the presence of 32 Niemann-Pick-like cells. Additionally, there was observed proliferation and abnormal localization of precursor cells, scattered T and B lymphocytes and plasma cells, a decreased ratio of granulocytes to nucleated red blood cells, and increased number of nucleated red blood cells. The presence of Gaucher cells was not detected. Immunohistochemical findings revealed no significant increase in the number of histiocytes. Screening for lysosomal accumulation disease showed a slight decrease in enzyme activity associated with Gaucher disease [glucocerebrosidase 1.17 µmol/L/h (normal range: 1.26 µmol/L/h to 22.23 µmol/L/h)]. After obtaining informed consent from the parents, peripheral blood samples were collected from the patient and his parents for trio whole-exome sequencing. The results of whole-exome testing revealed a heterozygous deletion variant of approximately 2.21 MB in the chr1:367658–2,577,002(p36.33p36.32) region, suspected to be located at a non-exact position in the child, while both his father and mother exhibited wild-type genotypes. An ACMG score of 1.05 was obtained, indicating the variant was pathogenic. The *GBA* gene associated with Gaucher disease showed a single heterozygous mutation, but there was insufficient evidence to confirm its pathogenicity, necessitating further examination for diagnosis.

**Fig. 1:**
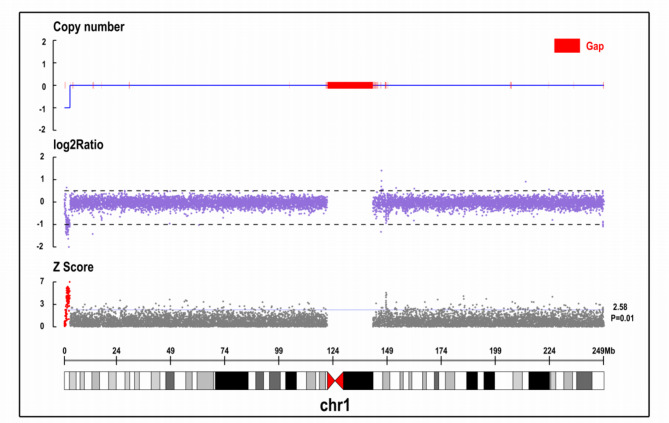
Results of genome copy number variation sequencing. Whole-genome sequencing (WGS) presented a heterozygous deletion of 2.58 Mb at position chr1:1-2580976. Copy Number: The number of copies calculated in this region: 0, 1, 2, -1, -2 indicate normal copy number, single duplication, double duplication, heterozygous deletion, and homozygous deletion. log2Ratio:log2 value of test/control, indicating the degree of difference compared with the control. Z score: The significance of the difference between the test and control quantities was calculated after the Z test; the larger the value, the more significant the difference between the test and the normal control

Considering that previous steroid therapy was effective and the condition relapsed after discontinuation, we opted to administer a high-dose steroid (2 mg/kg). On the seventh day, the blood cell count revealed a leukocyte count of 2.79 × 10^9^/L, a hemoglobin count of 92.0 g/L, and a platelet count of 50.0 × 10^9^/L. The patient was discharged on October 26, 2021, and commenced receiving regular steroid therapy. On December 1, 2021, the leukocyte count was recorded as 5.82 × 10^9^/L, the hemoglobin level was measured at 120.0 g/L, and the platelet count was documented at 89.0 × 10^9^/L. The changes in blood cell counts are shown in Table 1. The patient’s clinical phenotype of pancytopenia remained undiagnosed despite autoantibody and bone marrow laboratory examinations. The diagnosis of IRH was eventually confirmed due to the observed improvement following regular glucocorticoid therapy. On November 10, 2021, further whole-genome sequencing (WGS) presented a pathogenic heterozygous deletion of 2.58 Mb at position chr1:1-2580976 (Fig. 1).

**Table 1:** Change of blood cell count

time	white blood cell(×10^9^/L)	hemoglobin(g/L)	platelet(×10^9^/L)
August 24, 2021	0.67	112	1
September 18, 2021	5.47	121	42
October 19, 2021	0.90	86	39
October 25, 2021	2.79	92	50
December 1, 2021	5.82	120	89

## Discussion and conclusion

The chromosome 1p36 deletion syndrome, initially described by Yunis et al. [5], is a common subtelomeric microdeletion observed in humans, primarily resulting in dysmorphism and intellectual disability. The progress in cytogenetic techniques enables the identification of the connection between the clinical phenotype and the genetic cause of the syndrome. The most common manifestations observed in patients with 1p36 deletion syndrome include large fontanelle (100%), motor retardation/hypotonia (92%), moderate to severe intellectual disability (92%), growth retardation (85%) ), pointed chin (80%), eye/vision disorders (75%), seizures (72%), flattened nasal bridge (65%), slanted and/or short fifth finger (64%), low-set ears (59%), ear asymmetry (57%), hearing defects (56%), abusive behavior (56%), spiral thickening of the ear (53%), and deep-set eyes (50%) [6]. Jacquin C et al. reported 86 patients diagnosed with 1p36 deletion syndrome, who were further categorized into two groups based on the deletion position: 56 patients with distal deletions and 11 patients with more proximal deletions. The patients mainly presented with facial dysmorphism, microcephaly, developmental delay or intellectual disability, hypotonia, epilepsy, brain malformations, behavioral disorders, cardiomyopathy or cardiovascular malformations, and growth retardation. Cardiac abnormalities, brain malformations, and epilepsy appeared to be more frequent in Group distal deletions, whereas microcephaly seemed to be more common in Group proximal deletions. Developmental delay is a constant feature of the 1p36DS, in variable degree [7]. The classic phenotypes of this patient were intellectual disability, growth retardation and strabismus, without typical facial deformities. However, the abnormal phenotype of hematology in 1p36 deletion syndrome has rarely been reported, especially **IRH**. By searching chr1:1-2580976 in the DECIPHER database, we identified 279 variants exhibiting overlapping or identical deletion patterns. Subsequently, we screened for hematologic phenotypes and observed only one patient displaying hematologic abnormalities, manifesting as aberrant thrombocyte morphology and abnormality of blood and hematopoietic tissues. This patient, identified as number 401,654, presented a 3.16 Mb heterozygous deletion at position chr1:1:914087–4,071,190, while his matching CNV variation was only 1p36 deletion syndrome. Mosad et al. reported the association of 1p36.3 deletion with survival in patients with chronic HCV infection and type B non-Hodgkin’s lymphoma [8].1p36.11 has been reported as a novel risk locus for chronic lymphocytic leukemia. It can be inferred that the 1p36 deletion syndrome may be associated with bone marrow hematopoietic abnormalities.

The prediction of an individual’s phenotype based on the location and extent of 1p36 deletion remains a challenge due to the unidentified majority of genes responsible for 1p36-related phenotypes. Additionally, the haploinsufficiency of more than one gene may result in certain phenotypes. The atypical phenotypes of this patient might be associated with the involvement of specific genes located in this deletion chromosome region. Several genes closely-related with 1p36 include *MMP23B, GABRD, SKI, PRDM16, KCNAB2, RERE, UBE4B, CASZ1, PDPN, SPEN, ECE1, HSPG2, and LUZP1* [9].Deletion of the *KCNAB2* results in developmental delay, intellectual disability, and seizure symptoms [10]. Haploinsufficiency of *SKI* is thought to contribute to developmental delay, intellectual disability, epilepsy, orofacial clefts, and congenital heart defects [9]. Haploinsufficiency of *GABRD* has been suggested as a possible cause of neurodevelopmental abnormalities, neuropsychiatric problems, and seizures in children [11]. Gajecka et al. found that Haploinsufficiency of *MMP23B* led to large fontanelle with delayed closure, whereas overexpression led to premature closure of the cranial suture [12]. The whole-genome sequencing analysis of the patient found that the variant region covered genes containing *VWA1, PEX10, TMEM240, B3GALT6, GNB1, GABRD, ATAD3B, ATAD3C, MMEL1, ISG15, TNF RSF4, AGRN, SKI, DVL1, ATAD3A, MMP23B, and PRKCZ*. Among them, *GNB1* gene haploinsufficiency has been reported in the ClinGen database. The phenotype reported with *GNB1* variants included developmental delay hypotonia, seizures, ophthalmological anomalies, and persistent growth delay [13]. It should be noted that the G protein is a heterotrimer of α, β, and γsubunits which are encoded by respective members of the GNA, GNB, and GNG gene families. The G protein complex interacts with G-protein-coupled receptors (GPCRs), which are responsible for regulating vital cellular functions and cell proliferation [14].GPCR mutations, on the other hand, can cause a spectrum of disorders, including neurogenic disease, heart disease, metabolic disturbance, and hematologic disorders [13]. Previous research has reported that somatic mutations in *GNB1* and *GNB2* are associated with a spectrum of malignancies originating from both myeloid-derived and B-cell. Subsequent analysis showed reduced binding of the mutated subunits to Gαsubunits, resulting in increased signaling and downstream activity in cells expressing *GNB1* mutations, as well as cell growth. It may suggest the role of *GNB1/GNAS* in myeloid disease initiation [15].IRH is a bone marrow abnormality different from other known hemopoietic diseases and responds well to adrenocortical hormone and/or high-dose intravenous immunoglobulin treatment. Hui Liu et al. identified two autoantigens, the GPCR 156 variant and chain P, crystal structure of the cytoplasmic domain of human erythrocyte band−3 protein by LC-MS/MS in IRH. The study revealed that the GPCR 156 variant may play a role in suppressing the proliferation and differentiation of bone marrow hemopoietic cells by preventing binding with ligands due to structural changes, which could inhibit G protein activity [16]. The occurrence of IRH in our case may be linked to abnormalities in downstream targeting of GPCRs due to G protein beta of *GNB1* encoded subunit activation.

The clinical findings of the child were consistent with Gaucher’s disease, but the diagnosis of Glucocephalinase hypoplasia has not yet reached the diagnostic criteria, and the evidence of *GBA* pathogenicity by genome-wide testing is insufficient. The diagnosis necessitates additional examinations, including *GBA* enzyme activity assay and glucose sphingomyelin assay.

So far, there is extremely limited knowledge about hematology disorders in patients with 1p36 deletion. We represented a boy who lacked distinctive signs and symptoms of chromosome 1p36 deletion syndrome. However, He exhibited immune-related hemocytopenia that may be reported for the first time. Genotype-phenotype correlation may be helpful to localize the genes responsible for several clinical features of the syndrome. The identification of genes associated with specific phenotypic characteristics of the syndrome may facilitate more precise treatment of individuals with 1p36 deletion and enable the identification of mutations in monogenic diseases. Trio-based whole-exome sequencing has implications for precise cytogenetic diagnoses and accurate prognostic estimates.

## Methods

### Exome sequencing

Exome sequencing was carried out at Chigene (Beijing, China) according to standard diagnostic procedures.Before exome sequencing, participants gave written informed consent. In brief, genomic DNA was extracted from whole blood. Exome capture was captured and sequenced with IDT The xGen Exome Research Panel v2.0 whole exome capture chip. The genetic variants were analyzed by an in-house system and graded in conjunction with the Genetic Disease Clinical Characterization Database. After PCR of the target sequences, they were verified by Sanger sequencing on an ABI3730 sequencer, and the verification results were obtained by sequence analysis software.

### Genome sequencing

Genome sequencing was carried out at Chigene (Beijing, China) according to standard diagnostic procedures. Prior to exome sequencing, participants gave written informed consent. Genomic DNA was extracted from whole blood and sequencing libraries were constructed by random interruption methods. The constructed sequencing libraries were up-sequenced with no less than 99% genome coverage. Genetic variants were analyzed using a three-factor grading system as well as the ACMG genetic variant grading system.Possible structural variants were also annotated and analyzed.

## Data Availability

The datasets generated and/or analyzed during the current study are available in the [NCBI] repository, [SUB13179233].
